# Plasticity in organic composition maintains biomechanical performance in shells of juvenile scallops exposed to altered temperature and pH conditions

**DOI:** 10.1038/s41598-021-03532-0

**Published:** 2021-12-17

**Authors:** Nelson A. Lagos, Samanta Benítez, Cristian Grenier, Alejandro B. Rodriguez-Navarro, Claudio García-Herrera, Aldo Abarca-Ortega, Juan F. Vivanco, Isabel Benjumeda, Cristian A. Vargas, Cristian Duarte, Marco A. Lardies

**Affiliations:** 1 Facultad de Ciencias, Centro de Investigación e Innovación para el Cambio Climático (CiiCC), Universidad Santo Tomás, Ejercito 146, Santiago, Chile; 2 Instituto Milenio de Socio-Ecología Costera (SECOS), Santiago, Chile; 3 Programa de Doctorado en Biología Marina, Instituto de Ciencias Marinas y Limnológicas, Universidad Austral de Chile, Valdivia, Chile; 4 Departamento de Petrología y Mineralogía, Facultad de Ciencias, Universidad de Granada, Granada, Spain; 5 Departamento de Ingeniería Mecánica, Universidad de Santiago de Chile, Santiago, Chile; 6 Centro de Tecnología Biomédica, Universidad Politécnica de Madrid, 28223 Pozuelo de Alcorcon, Madrid Spain; 7 Facultad de Ingeniería and Ciencias, Universidad Adolfo Ibáñez, Viña del Mar, Chile; 8 Facultad de Artes Liberales, Universidad Adolfo Ibañez, Santiago, Chile; 9 Laboratorio de Ecosistemas Costeros y Cambio Ambiental Global (ECCALab), Facultad de Ciencias Ambientales & Centro EULA Chile, Universidad de Concepción, Concepción, Chile; 10 Departamento de. Ecología y Biodiversidad, Facultad de Ciencias de la vida, Universidad Andrés Bello, Santiago, Chile; 11 Centro de Investigación Marina Quintay (CIMARQ), Facultad de Ciencias de la Vida, Universidad Andrés Bello, Santiago, Chile

**Keywords:** Ecology, Ocean sciences

## Abstract

The exposure to environmental variations in pH and temperature has proven impacts on benthic ectotherms calcifiers, as evidenced by tradeoffs between physiological processes. However, how these stressors affect structure and functionality of mollusk shells has received less attention. Episodic events of upwelling of deep cold and low pH waters are well documented in eastern boundary systems and may be stressful to mollusks, impairing both physiological and biomechanical performance. These events are projected to become more intense, and extensive in time with ongoing global warming. In this study, we evaluate the independent and interactive effects of temperature and pH on the biomineral and biomechanical properties of *Argopecten purpuratus* scallop shells. Total organic matter in the shell mineral increased under reduced pH (~ 7.7) and control conditions (pH ~ 8.0). The periostracum layer coating the outer shell surface showed increased protein content under low pH conditions but decreasing sulfate and polysaccharides content. Reduced pH negatively impacts shell density and increases the disorder in the orientation of calcite crystals. At elevated temperatures (18 °C), shell microhardness increased. Other biomechanical properties were not affected by pH/temperature treatments. Thus, under a reduction of 0.3 pH units and low temperature, the response of *A. purpuratus* was a tradeoff among organic compounds (biopolymer plasticity), density, and crystal organization (mineral plasticity) to maintain shell biomechanical performance, while increased temperature ameliorated the impacts on shell hardness. Biopolymer plasticity was associated with ecophysiological performance, indicating that, under the influence of natural fluctuations in pH and temperature, energetic constraints might be critical in modulating the long-term sustainability of this compensatory mechanism.

## Introduction

Coastal areas experience large variations in temperature, salinity, oxygen and pH/*p*CO_2_ levels that have great influence on the performance of benthic organisms^1–3^. Off the Southern Pacific coast, regional (El Niño Southern Oscillation, ENSO) and global processes add up to seasonal, inter-annual and inter-decadal variability to the coastal ecosystems^4,5^. In addition, mesoscale processes such as coastal upwelling events show a wide range of variability in temperature, oxygen, and pH levels in very short time scales that have a great impact on animals inhabiting those ecosystems, especially calcifying invertebrates (i.e., mollusks, corals, barnacles, encrusting algae) which are vulnerable to these changes^6–9^. Coastal upwelling areas are characterized by seasonal, episodic events that bring subsurface waters of low temperature and low pH to shallower depths that may negatively impact benthic animals^9–12^. We now know that many organisms living in coastal upwelling areas either adapt to the local conditions^7^ or tolerate acute exposures to potentially stressful conditions^11^. Despite the low-temperature, low-pH upwelling conditions that generally persist in the coasts for periods of days, though more frequent and prolonged events due to increasing upwelling and favorable winds induced by global warming are expected for the following decades^13,14^, with potential impact on marine populations^15^.

The effects of temperature and pH/*p*CO_2_ on benthic organisms, mainly in the context of ocean warming and ocean acidification, have been extensively studied. Together they define key trade-offs between physiological processes, metabolic rates and shell calcification^16,17^, which also indicate the need for studies regarding the combined impacts of pH and temperature in the context of the whole organism^18^, and natural environmental variability^7,10^. However, the effects of environment-induced physiological compensations (due to changes in temperature and pH) on the shell structure and functionality of molluscan calcifiers (e.g., gastropods, bivalves) have been significantly less studied.

Marine molluscs have developed a broad diversity of shelled structures, the main function of which is to protect the organism against biological and environmental challenges (e.g., predators, waves forces). However, under high *p*CO_2_/low-pH conditions, the reduction of carbonate saturation state (Ω) favours the thermodynamic mineral solubility of calcium carbonate, thus increasing the cost of shell formation and calcification^19^. Nevertheless, several studies demonstrate that shell calcification can be maintained and even enhanced under reduced pH levels^16,20–23^, since marine calcifiers have a biological control over the calcification process^24,25^. Still, reduced seawater pH can affect shell properties, inducing mineral dissolution and changing the mineral organization and mechanical properties^22,26–30^.

Both changes in pH and temperature affect a suite of physiological processes (e.g., ingestion and metabolic rates), which also drives trade-offs that may alter shell calcification, structure, and functionality in mollusks. For instance, studies suggest that under low pH conditions, scallops maintain positive growth but at the cost of reducing the periostracum thickness and increasing the expression of functional molecules for biomineralization^31^, while gastropods show minor impacts on their metabolism, feeding, and shell growth rates, but trade-offs against changes in shell mineralogy^22^. Thus, mollusks may cope with low pH-induced dissolution by increasing protection with a thicker shell periostracum^24,32,33^, adjusting the amount and composition of organic material occluded within the shell mineral^16,34,35^, or altering shell mineral properties^22,27–29,33^. These studies suggest that shell properties may also be part of complex compensatory mechanisms to maintain overall performance and homeostasis when mollusks are exposed to low pH conditions. Increased temperatures can have similar impacts to low pH conditions in mollusk survival, growth, and development and temperature effects become exacerbated in combination with other concomitant stressors^35^. For instance, increasing temperature reduces shell integrity in *M. edulis* under limited food availability conditions, which may result from allocating energy from shell building to maintenance costs based on internal reserves^36^. However, in the context of combined impacts of temperature and pH, it is still poorly understood how biomineral and biomechanical properties of mollusk shells participate in these physiological compensations.

*Argopecten purpuratus*, is a native scallop species cultured in coastal embayment areas along the Southern Pacific coast of Peru and northern Chile, which are exposed to episodic events of coastal upwelling^9–12^. Empirical studies have suggested that winds inducing coastal upwelling could intensify at mid-latitudes in response to increased land-sea temperature differences driven by global warming^37^, while upwelling duration is also predicted to increase and lasting for several days^14^. This potential scenario could lead to scallop aquaculture sites being subjected to abnormally low temperature and pH conditions for periods longer than a week. Although, recent studies suggest that at high temperature (18 °C) and low pH (7.6), *A. purpuratus* show tradeoffs between positive growth and metabolism with biomineralization processes, which may result in local adaptation to natural low pH variability^31^, another study indicates that at low temperatures (14 °C), *A. purpuratus* showed increased shell dissolution and reduced growth rates when exposed simultaneously to low pH conditions (~ 7.7), which may have ramifications for the aquaculture of this species in the region^30^. Shell integrity and size are important ecological attributes with relevant implications in the thinning process performed by the aquaculture industry, when scallops are subjected to mechanical sieving, being segregated by size, with the objectives of reducing density, avoiding feeding interference, and thus reducing the time needed to reach market size^30^. Thus, in this study, we evaluated the effect of simultaneous changes in pH and temperature on juvenile *A. purpuratus* scallops, based on an orthogonal experimental design, incorporating the potential scenario of exposure to low temperature/low pH conditions over longer periods (> 2-week period), a potential future scenario in coastal upwelling areas at this latitude (Tongoy Bay, Northern Chile, 30° S). We specifically measured shell properties such as density, mineral composition, and organization, and associated biomechanical attributes.

## Results

### Environment, experimental scenarios, and seawater carbonate chemistry

**Figure 1:**
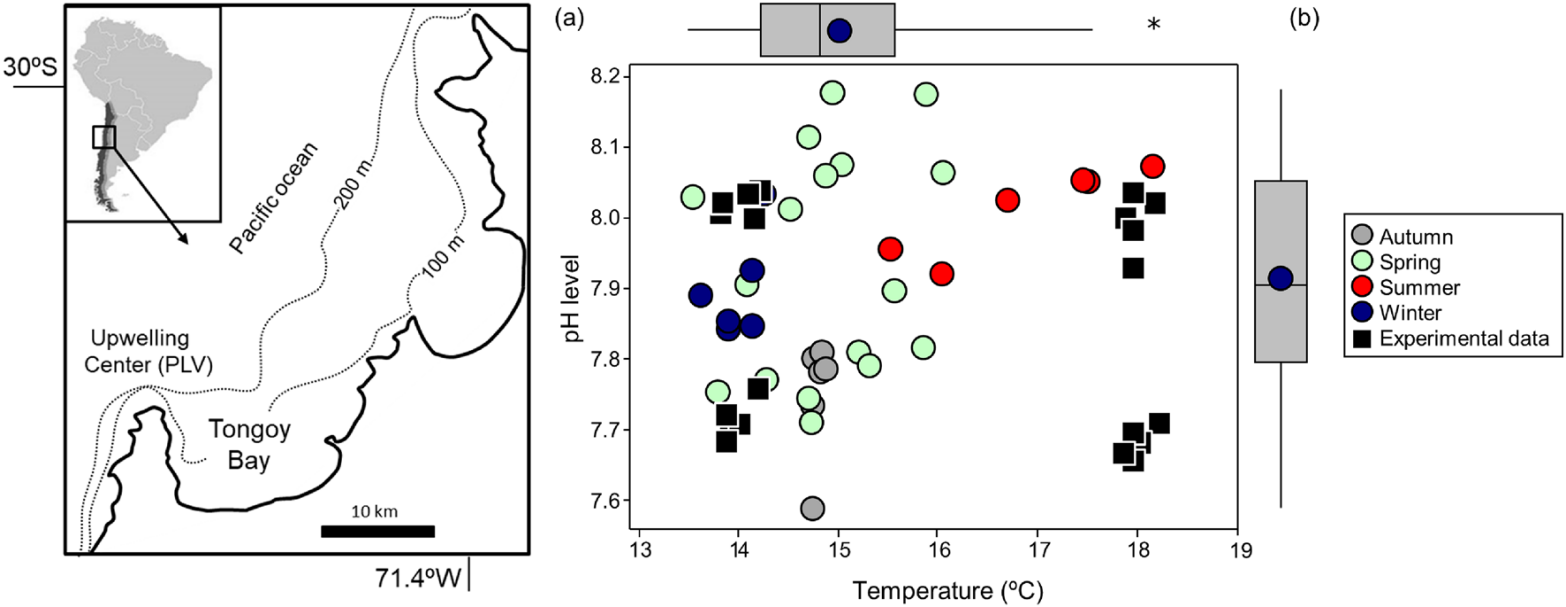
Environmental context of the study. **(a)** Tongoy Bay and Pt. Lengua de Vaca (PLV), the nearby upwelling centre influencing fluctuations in temperature and pH inside the bay where scallops *Argopecten purpuratus* are farmed; (**b**) Variability in pH and temperature for the combination of experimental treatments (solid black squares); overlapped with the natural variability in temperature/pH (solid circles) recorded seasonally inside Tongoy Bay from dec-2014 to May-2016. A boxplot (median, mean and range) for all records of temperature and pH are shown at the top and right side of the graph, respectively.

Environmental variation of Tongoy Bay evidenced the influence of upwelling condition at Lengua de Vaca point (Fig. 1a). Elevated temperatures and pH values (17–18 °C and pH 8.0) were found in summer, whereas low temperature/low pH waters (14–15 °C and pH 7.70–7.75) were observed mostly during upwelling events in spring season (Fig. 1b). These natural fluctuations overlap perfectly with our selected “*control scenario*” (pH ~ 8.0/14 °C) and “*upwelling condition*” scenario, respectively. For the field sampling period, only one (1.9%; n = 36) of the pH observations recorded inside the bay falls below pH = 7.7 (Fig. 1b). Similarly, only one temperature measurement was above 18 °C (1.9%, n = 36). Thus, except for the experimental treatment of high temperature and low pH, the resulting experimental orthogonal combination of temperature and pH values (Table 1), represented extreme, but realistic environmental variations occurring in Tongoy Bay (Fig. 1b). Low values of carbonate saturation state occurred at low pH scenarios, but all treatments showed saturated conditions for calcite (Ω > 1, Table 1), the calcium carbonate mineral polymorph precipitated by *Argopecten purpuratus.*

**Table 1:** Carbonate system parameters (mean ± SE) registered at each experimental treatment combining two nominal levels of pH (Control = 8.0 and reduced pH ~ 7.7) and temperature dominating in Tongoy Bay, Chile, during the experimental period (14 °C) and under warmer conditions (18 °C).

Carbonate System Parameter	14 °C		18 °C	
	pH ~ 8.0	pH ~ 7.7	pH ~ 8.0	pH ~ 7.7
Temperature (°C)	14.13 ± 0.19	14.08 ± 0.14	18.13 ± 0.11	18.14 ± 0.13
Salinity (PSU)	34.48 ± 1.78	35.86 ± 0.50	33.28 ± 1.97	34.00 ± 0.68
pH_NBS_	8.058 ± 0.017	7.754 ± 0.027	8.032 ± 0.041	7.720 ± 0.021
Total alkalinity (µmol kg^−1^)	1580.41 ± 206	1778.14 ± 127	1666.75 ± 117	1695.96 ± 133
*p*CO_2_ (µatm)	367.77 ± 62	891.26 ± 77	435.96 ± 74	969.48 ± 74
Ω_calcite_	2.07 ± 0.24	1.28 ± 0.13	2.29 ± 0.09	1.25 ± 0.13

*NBS* National Bureau of Standards.

### Shell morphology and microstructure

**Figure 2:**
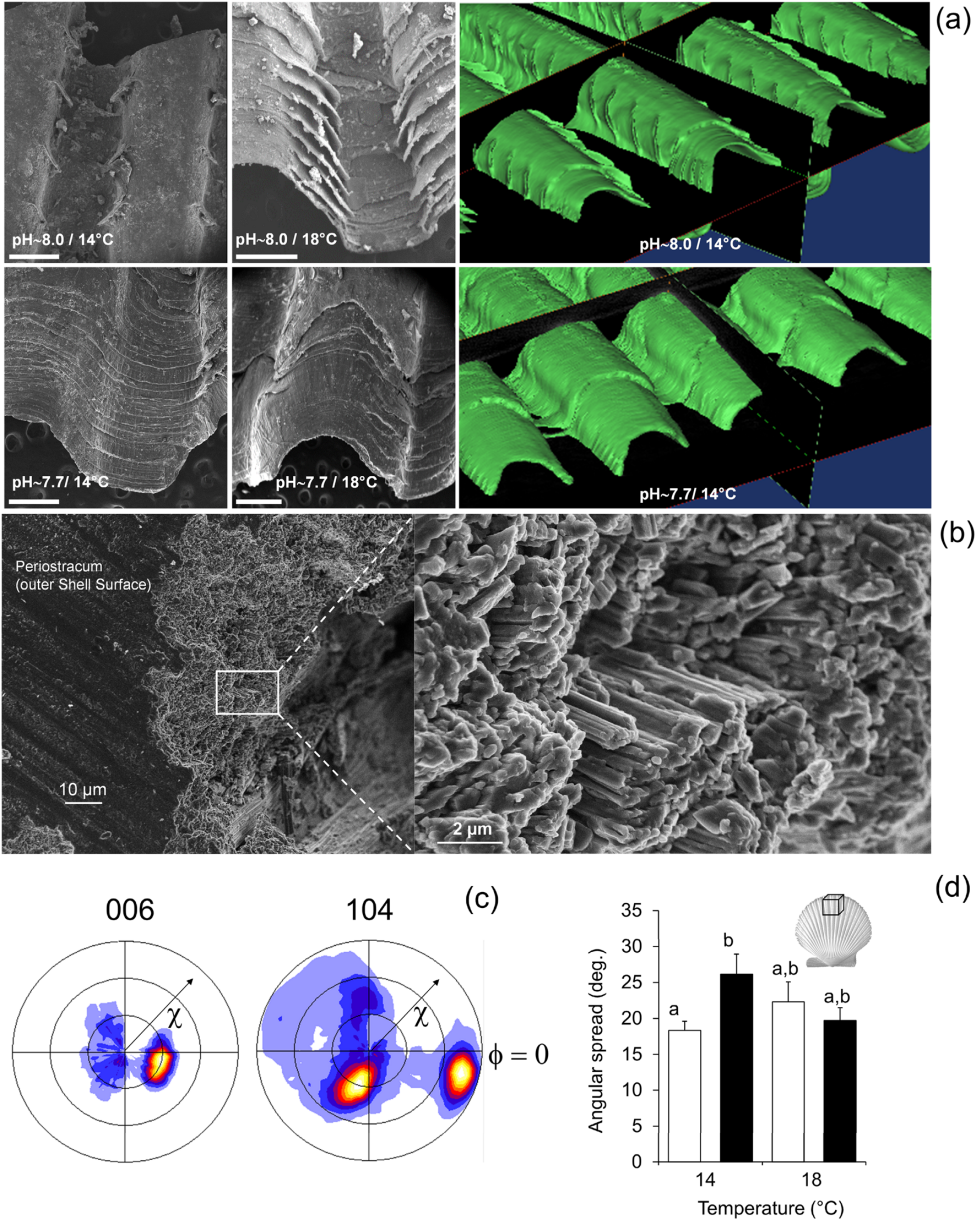
*Argopecten purpuratus* shell surface morphology (micro-CT and SEM images) showing the ribs of scallops exposed to control and reduce pH conditions (**a**). SEM images of the shell microstructure in a transversal fracture, showing bundles of co-oriented calcite crystal fibers (**b**); The 006 and 104 pole figures (determined by 2D-XRD^38,39^) display the 3D orientation of the c-axis and rhombohedral faces of calcite crystals making the shell (**c**). Angular spread of the calcite crystal (determined from 104 Gamma scans), showing the scattering of the orientation of crystals in the shell outer surface (**d**).

*A. purpuratus* shells have conspicuous ribs that radiate from the shell umbo. The outer shell surface is decorated by steps or terraces advancing parallel to the shell margin and that are associated to growth events. However, SEM and Micro-CT images showed that these structures are only observed in individuals raised at higher temperature conditions and control pH levels and absent under low temperature and low pH conditions (Fig. 2a). The shell mineral at the growing edge of the scallop shells is made of calcite crystal fibers of 1–2 microns thick, are arranged in parallel bundles, ending in well-defined rhombohedral faces with their elongation axis forming an angle to shell surface (Fig. 2b). Analysis of calcite crystal orientation by XRD confirm that crystals are highly aligned. The 006 pole figure displayed one maxima that is offset ca.30 degrees from the center (Fig. 2c), indicating that calcite crystals have their *c*-axis aligned but tilted 30 degrees from the shell toward the shell edge. In addition, the 104 pole figure showed three maxima around the position of the 006 maxima (i.e., the *c*-axis) that mark the disposition of {104} rhombohedral faces according to the three-fold symmetry of calcite (Fig. 2c). Thus, *A. purpuratus* shells have a fibrous microstructure of well-ordered calcite crystals with their three crystallographic axis co-oriented, though there is a significant scattering in the orientation of crystals (ca. 20–30 degrees), as indicated by the angular spread of maxima displayed in the pole figures or that measured in the 104 Gamma scans. When comparing the angular spread of calcite crystals from individuals grown at different conditions, the specimens at reduced pH conditions showed a significant increased angular spread (lower crystal orientation) (Fig. 2d). However, at higher temperature, there were no differences (Table 2) indicating that the effect of pH might be ameliorated at increased temperature (Fig. 2d).

**Table 2:** Summary results (2-way ANOVA) for shell mineral (TGA) and periostracum (ATR-FTIR) organic composition, and crystal orientation (XRD). These properties were measured at the growing shell edge of *Argopecten purpuratus* scallops.

Analytical Technique	Shell properties	df (source, error)	Temperature (T)		pH level		T × pH		Interaction coefficient
			F	*p*	F	*p*	F	*p*	
TGA	Water content	1, 19	3.78	0.070	11.26	**0.004**	0.68	0.442	
	Organic matter Phase-1	1, 19	5.19	**0.037**	4.08	0.060	6.38	**0.022**	− 0.0019
	Organic Matter Phase-2	1, 19	2.14	0.163	1.90	0.187	0.14	0.709	
	CO_2_ Loss	1, 19	0.88	0.363	7.44	**0.015**	0.00	0.975	
	Total Organic Matter	1, 19	9.80	**0.006**	1.34	0.264	7.11	**0.017**	− 0.0016
ATR–FTIR	Sulfates	1, 19	1.53	0.235	7.89	**0.012**	3.23	0.091	
	Polysaccharides	1, 19	9.51	**0.007**	12.88	**0.002**	7.09	**0.017**	+ 0.0019
	Proteins (amides)	1, 19	0.46	0.508	4.27	**0.055**	3.29	0.089	
	Carbonates (CO_3_)	1, 19	6.63	**0.020**	9.40	**0.007**	4.32	0.054	
	Lipids	1, 19	0.17	0.686	0.18	0.679	0.02	0.896	
	OH + Amides A	1, 19	1.70	0.210	0.14	0.711	0.27	0.611	
XRD	Crystal Orientation	1, 19	0.27	0.611	1.24	0.282	5.37	**0.035**	− 0.0270

F and *p* are F-ratio and *p*-values, respectively. Significant *p*-values (*p* < 0.05) are shown in bold. The coefficient identifies antagonistic (−) and synergistic (+) effects in the interaction of both environmental variables.

### Shell mineral and periostracum composition

**Figure 3:**
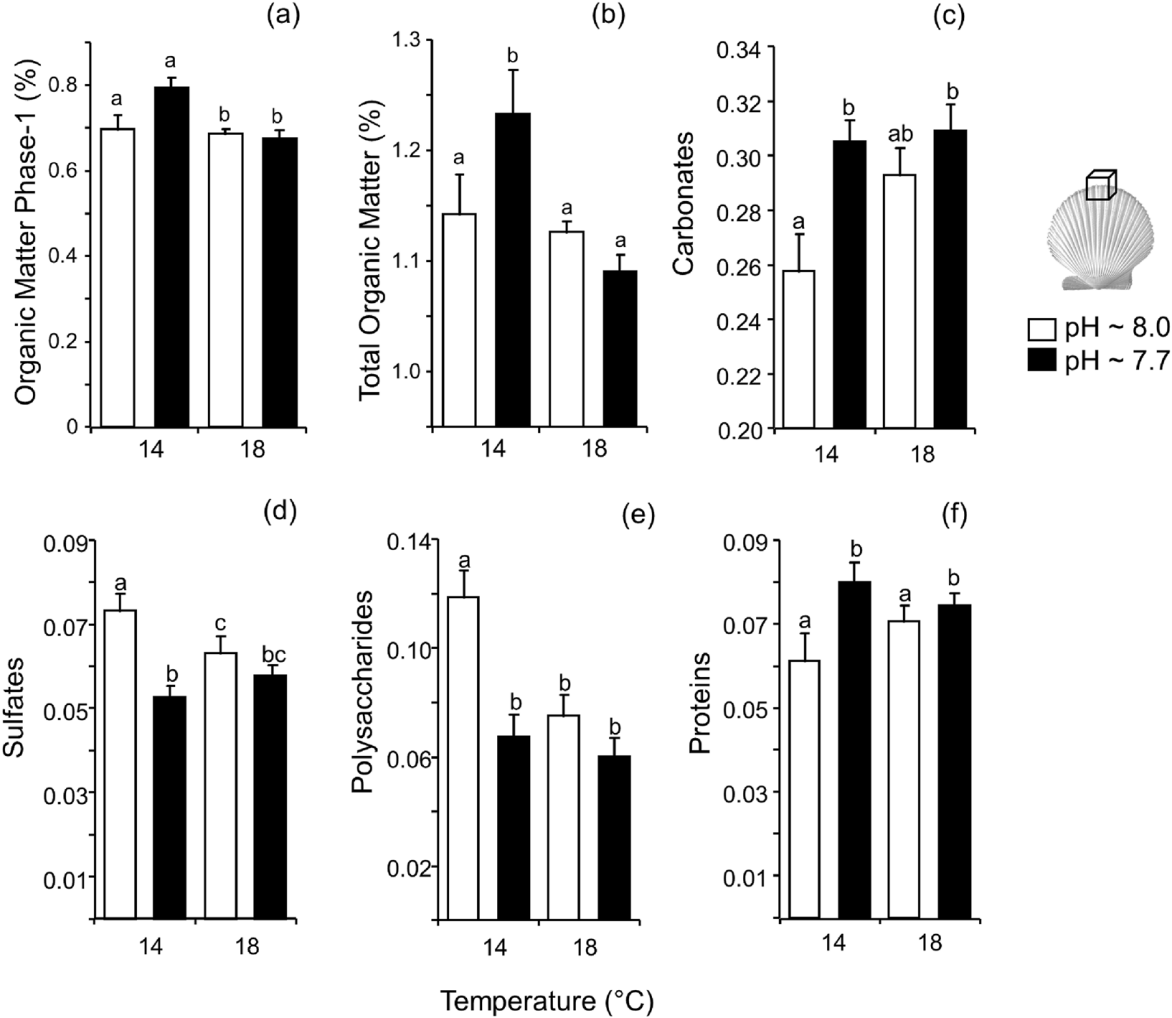
(**a**,**b**) Influence of temperature and pH on *Argopecten purpuratus* shell mineral composition (organic matter determined by TGA, % Mean ± SE); and (**c**–**f**) shell periostracum composition (determined by ATR-FTIR normalized signal, mean ± SE) Different letters represent significant differences among treatments using a post hoc Tukey HSD Test.

The total and inter-crystalline organic matter (OM phase-1) content in the *A. purpuratus* shell mineral decreased significantly at warmer conditions, with no significant differences between pH levels (Fig. 3a,b), and both variables showed a significant antagonistic effect (Table 2). SEM observations showed that the shell periostracum was a thin fibrous organic layer coating the outer shell mineral surface, but that was irregularly distributed and even eroded under low pH conditions in both temperature treatments (Supplementary material, Figs. S1–S4). Analysis of the periostracum coating the outer shell surface by infrared spectroscopy showed an increase in the carbonate signal at reduced pH conditions in both temperature levels (Fig. 3c) indicating that the periostracum is thinner in these experimental treatments. Sulfates and polysaccharides decreased significantly at reduced pH levels and increased at higher temperatures (Fig. 3d–e), which in the case of polysaccharides lead to a significant and synergistic interactive effect between pH and temperature (Table 2). On the contrary, proteins showed a significant increase under low pH conditions, independent of temperature variations (Fig. 3f). Other bands associated with OH group and lipids on the shell periostracum of *A. purpuratus* showed no statistical differences among treatments (Table 2).

### Shell density and biomechanical performance

**Figure 4:**
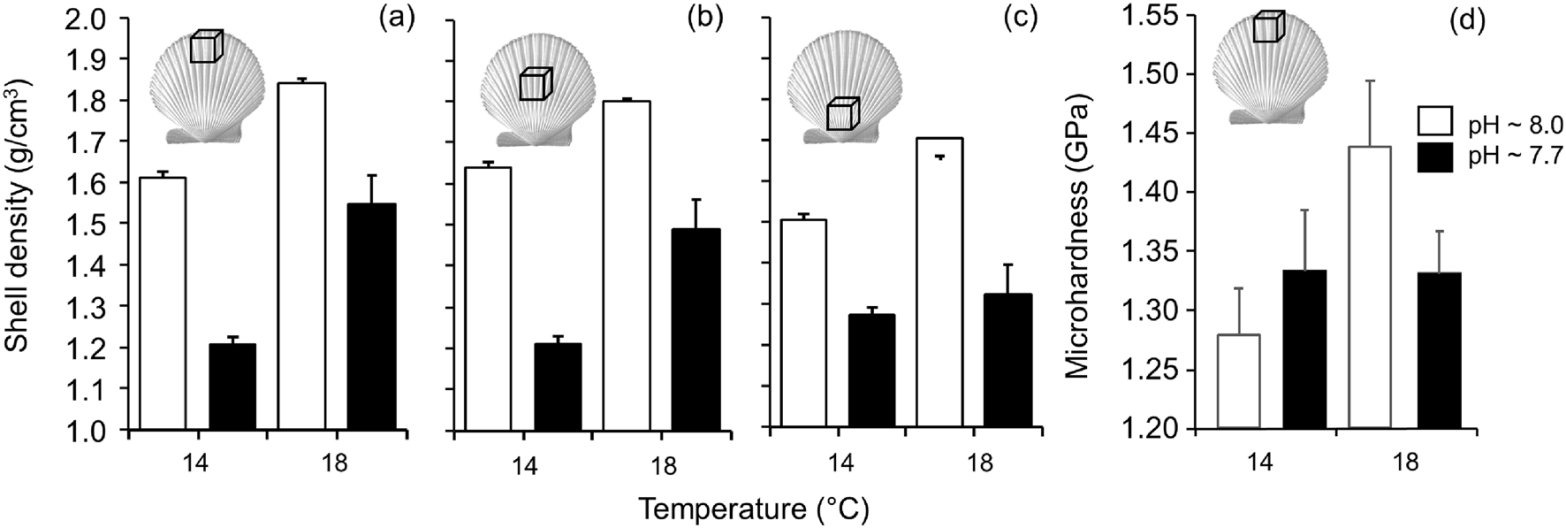
Influence of temperature and pH on *Argopecten purpuratus* shell properties: (**a**–**c**) Shell density (mean ± SE) (determined by Micro-CT) at three different regions of the shell; (**d**) micro-hardness (determined by micro-indentation) at the shell growth edge. Different letters in (**a**) represent significant differences among treatments using a post hoc Tukey HSD. Posteriori test was not performed in (**a**–**c,e**) because the interaction of random with fixed effects.

**Figure 5:**
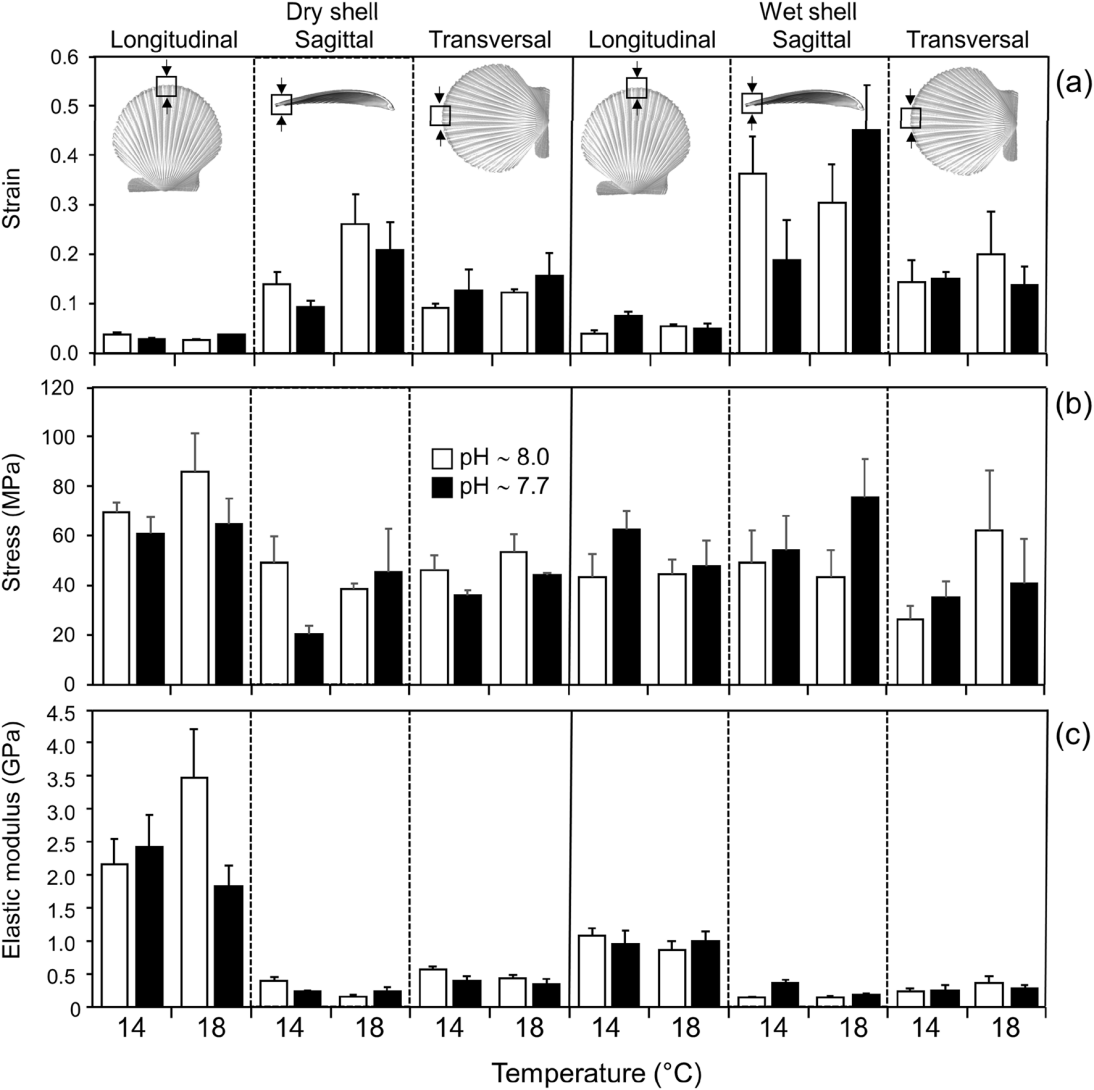
Influence of temperature and pH on *Argopecten purpuratus* shell biomechanical properties. Compression test (mean ± SE.) performed on *A. purpuratus* at the growing edge of the shells under dry and wet conditions and along three main orientation planes of the shell.

Shell density showed a significant reduction at low pH conditions (*F*_(1, 4)_ = 55.03, *p* = 0.002) with an effect reversal at warmer conditions (*F*_(1, 4)_ = 21.03, *p* = 0.010), but the interaction between treatments was not significant. However, though overall higher shell density was recorded in animals exposed to increased temperatures shell density was reduced at the central and anterior (umbo) regions of the scallop shells (*F*_(4, 48)_ = 3.83, *p* = 0.009; Fig. 4a–c). Microhardness of *A. purpuratus* shells increased under warmer conditions (*F*_(6, 124)_ = 3.63; *p* = 0.002), with no effects of pH (Fig. 4d). In addition, strain or deformation of the shell material was affected by the interaction of shell condition (wet/dry) with orientation of compression test (Fig. 5a, F_(2,86)_ = 4.18; *p* = 0.018). Wet shells and compression across the sagittal plane (shell thickness) showed increased strain respect to dry shells (*F*_(1, 86)_ = 11.49, *p* = 0.001) and longitudinal axis (*F*_(2, 86)_ = 34.14; *p* = 0.001). Mechanical stress was variable between shell condition and orientation (*F*_(2, 86)_ = 4.93; *p* = 0.009), but evidencing a significant increase in stress along the longitudinal axis (*F*_(2, 86)_ = 4.66; *p* = 0.012) (Fig. 5b). The elastic modulus (stiffness) showed a significant interaction between shell condition and orientation (*F*_(2, 86)_ = 27.42; *p* < 0.001). Dry shells showed higher stiffness than wet shells (*F*_(1, 86)_ = 41.23; *p* < 0.001), and mostly along the longitudinal axis (*F*_(2, 86)_ = 144.42; *p* < 0.001); elevated temperature increased shell strain (*F*_(1, 86)_ = 4.53; p = 0.036) and stiffness (*F*_(1, 86)_ = 4.43; *p* = 0.038), but no effect of pH levels were observed (Fig. 5c).

### Physiological rates and organic composition correlations

**Figure 6:**
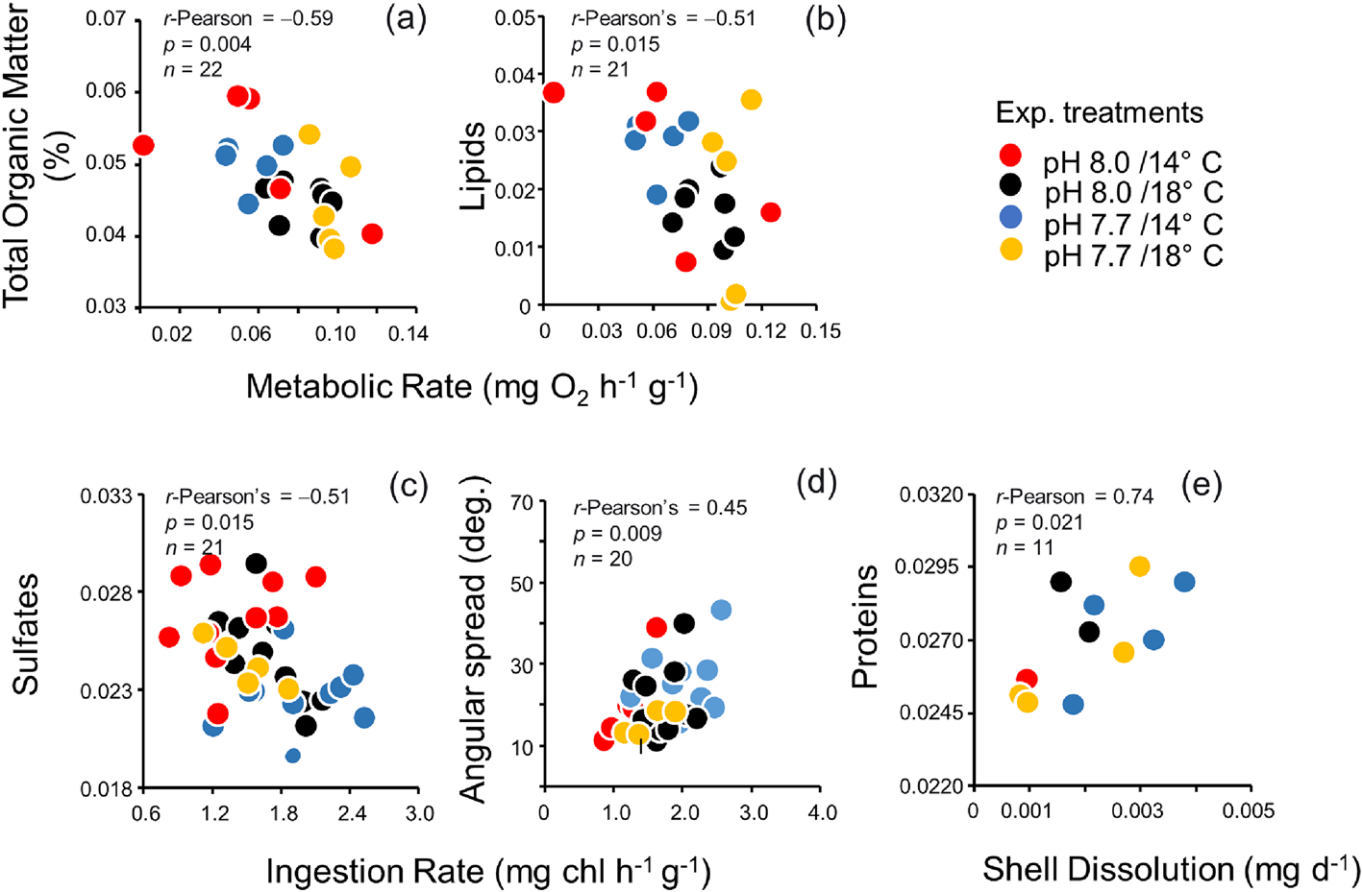
Significant correlations (*r*-Pearson) between (**a**) total organic matter in the carbonate matrix of *Argopecten purpuratus*; (**b**,**c**,**e**) organic compounds, and (**d**) calcite crystal angular spread recorded in the shell periostracum with metabolic(**a**,**b**), ingestion (**c**,**d**) and dissolution rates (**e**) (see also Table S1 supplementary material). Dots of different color indicate different combinations of pH/temperature treatments.

Physiological traits and shell dissolution previously recorded in *A. purpuratus* subjected to pH/temperature treatments (Table S1 in supplementary material, Lagos et al., 2016, Lardies et al., 2017) were correlated with variations in the organic content in the shell mineral and specific periostracum chemical components evaluated on the same scallop individuals (Fig. 6). Both total organic matter (Pearson–*r* = − 0.59, *p* = 0.004) and lipids (*r* = − 0.51, *p* = 0.01) were negatively correlated with metabolic rates recorded in the same scallops (Fig. 5a,b). On the contrary, ingestion rate was negatively correlated with shell sulfate content (*r* = − 0.38, *p* = 0.02), but positively correlated with the angular spread of calcite crystal (*r* = 0.33, *p* = 0.05) (Fig. 6c,d). Finally, the dissolution rate recorded in dead shell was positively correlated with the normalized signal of proteins remaining on the shell periostracum (*r* = 0.74, *p* = 0.02, Fig. 6e). However, these significant correlations between shell properties recorded in the same individual scallops must be interpreted with caution due to potential increased type 1 errors.

## Discussion

Shell integrity is critical for the survival of marine calcifiers, and this study demonstrated that the scallop *Argopecten purpuratus* maintains shell biomechanical properties under low pH and low temperatures. Present results, along with previous studies done on the same individuals (see Table S1 in supplementary material), suggest that energetic demands of these physiological rates negatively impact the amount and composition of organic compounds deposited in the shell mineral matrix and surface periostracum of scallops. Our results reveal that organic content in the shell mineral and periostracum and calcite crystallographic orientation of *A. purpuratus* shells are plastic and correlated with variations in pH and temperature^16,33^. Low pH increased the amount of organic matter occluded within the shell mineral and altered the organic composition of the shell periostracum, increasing its protein content but reducing sulfates and polysaccharides. Regardless of temperature, low pH exposure increased the carbonate signal at the shell outer surface, which is indicative of a thinner periostracum^16,31,34^. A thinner periostracum may increase the exposure of the shell minerals, making them more susceptible to dissolution, affecting shell integrity and functionality. However, our biomechanical data (mechanical strain, stress, and elastic modulus) show that these environmentally induced changes in shell composition did not compromise shell biomechanical functionality, with exposure to high temperatures even improving shell density and microhardness.

Several studies have reported that under low pH conditions, the synthesis of biomineralization molecules (e.g., chitin) become stimulated, which may lead to increased shell growth^31,40,41^. However, other studies have reported decreased content of organic matter in mussel shells at low pH levels, but which is ameliorated under warmer conditions^42,43^. Our results show that at low pH and low temperature conditions, the periostracum protein content increases whereas sulfated polysaccharides content decreases. Similar results have been found in mussels exposed to natural low pH conditions^16^. However, in juvenile *A. purpuratus* scallops under stressful conditions of acidification (low pH) and low food supply, there are changes in the periostracum with an increase in polysaccharides production while proteins and lipids remain unaltered^31^. In addition, we found that the increase in proteins occur only under low pH and in combination with elevated temperature; conditions that are accompanied by a reduction in periostracum thickness as deduced by the increase in carbonates band intensity. In mussels, the carbonate band intensity is proportional to the periostracum thickness^31,34^. Thus, it may be suggested that in the case of *A. purpuratus*, the reduction in periostracum thickness results from a reduction in sulfated polysaccharides synthesis. In fact, shell dissolution of *A. purpuratus* increased significantly under low pH and increased temperatures conditions^30^ and in the present study we found that dissolution rate in dead shell was positively associated with protein content, suggesting that this might be an indirect effect of the reduced periostracum deposition. These results also suggest the protective role of sulfated polysaccharides component of the periostracum shell. Thus, under low pH and both temperature conditions, the response of *A. purpuratus* was to tradeoff the increase of proteins deposited in the periostracum to maintain positive shell growth, but at the cost of reducing sulfated polysaccharides production, which might compromise the protective role of periostracum against dissolution of shell carbonates. The expression of functional molecules associated with biomineralization such as chitin synthase may be underlying these changes in proteins content under low pH conditions^31^. This plasticity and trade-offs in organic compounds (i.e., biopolymers plasticity) of *A. purpuratus* scallop shells may represent an additional compensatory mechanism to confront environmental stress and maintain shell functionality in terms of structural support, protection and/or absorption of impact energy^22,33^

Low pH conditions also alter the shell density of *A. purpuratus*. Recent studies using micro-CT techniques demonstrate that gastropods exposed to low pH conditions can lose ca. 40% of the shell density^46^. Both shell density and porosity are relevant (but inverse) indicators of mechanical strength, which under low pH conditions, have shown variable impact on shell fragility^45^. In our study, we found ca. 20% reduction in shell density due to low pH conditions, and this reduction occurred in both newly deposited and non-growing shell regions. Previous studies highlighted that acidification, in addition to reducing shell precipitation, may increase dissolution at non-growing or older shell regions (e.g., apex or umbo)^47^. These changes suggest increased fragility of the whole shell. Thus, changes in shell density of *A. purpuratus* evidenced that low pH can increase the vulnerability to durophagous and bioeroders predators reducing survival opportunities in mollusks^33,48^. However, our results also show that increased temperatures ameliorate these impacts suggesting a relevant role of temperature conditions in maintaining shell density.

The influence of moderate warming (i.e., + 4 °C) ameliorates the impacts of low pH on molluscan physiology (e.g., survival, growth, and development)^35^. In addition, we found that the combined effect of acidification and higher temperature operated antagonistically changing the mineral organization (crystal orientation) of *A. purpuratus* shells and increasing crystal disorder, thereby increasing shell density. Similar results have been described in mollusks exposed to low pH conditions^27–29^, but these effects on biomineralization may disappear under dramatically increased temperatures^44^ (i.e., far over the 4 degrees used in this study) These alterations in crystal orientation, may allow more organic matter to precipitate, as observed under low pH conditions^45^. It has been suggested that low pH induces a progressive disorder in crystal orientation in mussels, which is inversely associated with the concentration of polysaccharides in their shell periostracum^34^. Thus, reduction in polysaccharides of the periostracum (and increased carbonate exposure), may affect the biomineralization and altering the crystal organization. Several studies report that the orientation of calcite crystals can be determined by an oriented nucleation on the organic matrix sheets that have molecular groups matching the disposition of ions (Ca^2+^) in the mineral surface (i.e., pseudo-epitaxis^49,50^), and the organic periostracum can also act as a substrate or template for oriented nucleation of calcite crystals^51^. Thus, changes in the crystal orientation of *A. purpuratus* shells suggest that some fundamental mechanisms of the mineralization may become impaired (i.e., epitaxial nucleation). These modifications in shell density and crystallography of *A. purpuratus* shells represent further evidence of *mineral* plasticity, suggested as a compensation mechanism to maintain biomechanical stability and functionality of mollusk shells^22^. This compensation mechanism is in agreement with our results, indicating no biomechanical weakness of *A. purpuratus* shells when exposed to the combined influence of low pH and temperature.

In general, it is suggested that the shell strength in scallops is a function of shell height, thickness, corrugation, and convexity of the whole shell^52^. In mollusks, experimental and natural low pH conditions alter shell size and thickness^30,32,53^, and several studies reported that low pH reduces the shell breaking strength in oysters^54^ and mussels^40,55^. Recent studies showed that gastropods produced tougher shells under low pH conditions, while the elastic modulus was maintained^17^ and that high temperature can ameliorate these impacts on shell mechanical properties^54,55^. Our results partially agree with these finding because *A. purpuratus*, independent of pH conditions, produce a tougher shell at increased temperatures while the elastic module, strain and stress remained unaltered. In addition, the organic matter provided successful elasticity and deformation^45^, despite the low proportion in the scallop shells (~ 1%), which, jointly with trade-offs between proteins and polysaccharides/lipids, could be responsible for maintaining shell mechanical strength. Nevertheless, biomechanical test evaluation proved to be quite complex and shows the strong influence of shell conditions (wet/dry) and the direction of the compression tests, which might prevent determination of differences in these shell biomechanical properties between experimental groups^56^. Thus, biomechanical responses of mollusk shell may be highly variable, from negative effects on the shell strength^31,54,55,57^ to non-effects in gastropods^17,22^ and scallops (this study). Our results also indicate that variations in biomechanical responses of *A. purpuratus* shell could arise from variations in the biomaterial properties used to build different structural parts of the shell and orientation of measurements (i.e., anisotropy). For instance, shell stiffness increased along the shell ribs, which is also parallel to the growth of calcite crystals. Whether these variations depend on the hierarchical structure of the shell (i.e., micro-, nano-scales^45^), the building materials, shell geometry, shell density or others whole-shell properties, deserves more comparative studies specially to understand the interaction of climate stressors, shell structural changes and how these affect their biomechanical properties.

Finally, metabolic and feeding performance of *A. purpuratus* scallops is elevated at increased temperature^11^ and these traits were negatively associated with alteration in organic matter of the shell mineral organic matrix, periostracum chemical composition and shell mineral organization (calcite crystal orientation). These relationships show how physiological processes associated with energy acquisition may interplay with process modulating shell biomineralization. Additionally, associations with physiological traits evidenced that the structural and mineral components and biomechanical performance of scallop shells are part of the overall compensations occurring when mollusks confront stress conditions imposed by low pH and temperature fluctuations. Environmentally-induced changes in shell and periostracum organic composition (i.e., biopolymers plasticity), shell density and mineral organization (i.e., mineral plasticity^22^) seem the most plausible mechanisms used by *A. purpuratus* to maintain a functional shell and therefore confront environmental and biological threats. However, the lack of effect of elevated temperature on shell resistance and its positive effect on scallop shell hardness must be interpreted with caution, since elevated temperature (18 °C) conditions are extreme events, rarely occurring inside Tongoy Bay. Future predictions suggest an increase in upwelling intensity and duration at mid and high latitudes^14^. Although, the driving mechanisms are not clear, coastal upwelling in eastern boundary current systems have intensified and it seems that the increasing trend will continue^13,37^. Thus, persistent upwelling-induced low temperature and low pH conditions represent a potential threat for the structural composition and mineral density of scallop shells, imposing restrictions on the physiological performance of the whole organism in field conditions^12^. Further studies are required to establish if this biopolymer and mineral plasticity in *A. purpuratus* shells can be sustained over ecologically relevant time (months-years) and under the persistent influence of coastal upwelling. Our results highlight the crucial importance of monitoring upwelling-associated carbonate systems parameters in the aquaculture industry^12,58^ and the potential impacts of carbonate chemistry on shell strength of both wild populations and farmed scallops inside Tongoy Bay. Considering the high variability in the production of this valuable resource^30^, these results could contribute to the better management and adaptive capacity of this aquaculture activity.

## Methods

### Environment, experimental scenarios, and seawater carbonate chemistry

Juvenile *Argopecten purpuratus* scallops (~ 41 mm ± 1.3 SD shell length) were collected from Invertec—Ostimar Co. scallop farm located in Tongoy Bay (30° S, central Chile, Fig. 1a). Scallops were transported in a thermobox (ca. 14 °C) to the Calfuco Coastal Laboratory (Valdivia, Chile), kept for acclimation during 3 days under running seawater (13−14 °C), natural photoperiod, and fed daily with microalgae (*Tetraselmis* spp., ~ 65 × 106 cells ml^−1^). To examine the combined effects of temperature and pH, an orthogonal experimental design was considered by incorporating four experimental treatments, where three of these treatments were based on natural environmental conditions observed in Tongoy Bay: (1) Control condition (*non-upwelling*): 14 °C/pH ~ 8.0; (2) Low temperature/low pH (*upwelling condition*) (14 °C/pH ~ 7.7); (3) High temperature/high pH (*summer condition*) (18 °C/ pH ~ 8.0) and (4) an *artificial scenario* of high temperature/low pH condition (18 °C/pH ~ 7.7), which is not possible to observe at the temporal scale of the present study (upwelling cycle of days/week), but possible upon long-term climatic projections for this region^59^. Each treatment was replicated five times (5 aquaria) and each replicate contained four scallops. Bee tags were used to identify and track individual scallops during the experiment. All these experimental processes and animal manipulations were performed in accordance with guidelines and regulations established by the Chilean law.

We used a semi-automatic CO_2_ equilibration system to obtain selected low pH scenarios. Blended dry air was generated by compressing atmospheric air (117 psi, oil-free compressor) with pure CO_2_ using mass flow controllers (MFCs, AALBORG); this blend was then bubbled into experimental aquaria (replicates) and head tanks reaching *p*CO_2_ ~ 900 μatm in seawater^60^. Briefly, the *p*CO_2_-induced acidification ranges from 367 to 435 μatm for the control scenario, and 891–969 μatm according A2 emission scenario for the year 2100^61^, which yield nominal pH–levels of 8.0 and 7.7 units. These two pH treatments were combined in a full factorial experimental design with two temperature treatments (14 °C and 18 °C). The scallops were exposed for 18 d, since one of the main focuses of our study was determine how scallops could respond to prolonged low temperature/low pH conditions, upon a scenario of extended exposure to *upwelling condition*, which typically last from 2 up to 8 days at present^12,62^. Over the experimental period, temperature, salinity, pH_NBS_ and total alkalinity (AT) were monitored every 3 days to estimate the rest of carbonate system parameters (i.e., *p*CO_2_, calcite saturation state Ω) inside the experimental aquaria. Additionally, in field conditions we monitored seasonal variability of carbonate system variations inside Tongoy bay from December 2014 to May 2016. Discrete shipboard sampling of temperature was conducted at 2 and 8 m depth, using a SeaBird SBE-19plus CTD profiler. Additionally, discrete water samples for pH estimates were collected and measurements were standardized to total scale (pH_T_) by using a METROHM 713 pH meter (input resistance > 1013 Ohm, 0.1 mV sensitivity, and nominal resolution 0.001 pH units) using a glass combined double junction Ag/AgCl electrode (METHROM model 6.0219.100) calibrated with 8.089 Tris buffer 25 °C following DOE potentiometric method^10,60^.

### Shell morphology and microstructure

Shell pieces samples fractured from the growing shell edge were cleaned with deionized water and then carbon-coated (HITACHI UHS evaporator) prior to observations using Auriga CrossBeam Workstation operated at 5 kV (ZEISS, SEM). Crystallographic orientation was measured in fragments collected from the shell edge (ca. 3 × 3 mm, *n* = 5 per treatment, 1 per aquaria) using X-ray single-crystal diffractometer equipped with a CCD area detector (D8 SMART APEX, BRUKER, Germany). Working conditions of diffraction measurements were Mo Ka (0.7093 Å), 50 kV and 30 mA, a pin-hole collimator of 0.5 mm in diameter, and an exposure time of 20 s per frame. To quantify the orientation of crystals from 2D-XRD patterns or frames, the width of the peaks displayed in the intensity profile along 104 rings of individual frames (104 Gamma scan), which represent the angular spread or scattering in the tilting of the *c*–axis of calcite crystal within the shell mineral, was measured^34^. In selected samples, a set of 2D-XRD patterns (frames) was registered while rotating the sample around Φ angle (every 5 degrees). Pole figures displaying the three-dimensional distribution of specific crystallographic directions of calcite crystals (i.e., 006, 104,) were determined using XRD2DScan software v.7.0^38,39^.

### Shell mineral and periostracum composition

After the experiment, shells were washed with deionized water, oven dried at 60 °C for 4 h and small pieces (ca. 2 × 2 mm) were cut from the growing shell edge. *A. purpuratus* shell organic content was analyzed by Thermo-Gravimetric Analysis (TGA, METTLER-TOLEDO DSC1, Zurich, Switzerland). Pieces of the growing edge (*n* = 5 per treatment, 1 per aquaria), were powdered and ca. 25 mg was used for the analysis (25–900 °C; 20 °C min^-1^ heating rate). Main weight loss events occurred in the range of 20–180 °C due to loss of residual water; then to the combustion of inter– (ca. 180–400 °C; OM phase-1) and intra– (400–600 °C; OM phase-2) mineral organic matter (OM), and finally at temperatures > 600 °C to the CO_2_ loss due to thermal decomposition of carbonates^31,34^. Shell periostracum chemical composition was determined by infrared spectroscopy using Fourier Transformed Infrared Spectrometer (FTIR) with Attenuated Total Reflection (ATR) unit (FTIR model 6600, ATR Pro, JASCO, Japan) as described in more detail elsewhere^31^. Briefly, the outer surface of shell samples (*n* = 5 per treatment) randomly collected from the growing margin were pressed against the ATR window, and reflectance spectra were recorded at a 2 cm^−1^ resolution for 50 scans. The relative amounts of water, proteins, sulfates, carbonates, polysaccharides, and lipids, among others, were estimated from the absorption peak areas associated to each chemical component (e.g., O–H: water; C–H: lipids or fatty acids; amide bond: proteins; C–O: carbonates; S–O: sulfates; COC: sugars/polysaccharides), and then normalized to the total area of the FTIR spectra to provide a semiquantitative analysis.

### Shell density and biomechanical performance

Shell density (g/cm^3^) was evaluated (n = 5 per treatment) using Micro-Computed Tomography scanning (Micro-CT Skyscan 1278, BRUKER, Belgium). We use 300 two-dimensional scans per sample, and then convert into a 3*D* reconstructed model (CT-Vol software v. 2.3.2.0; https://www.bruker.com/en/products-and-solutions/microscopes/3d-x-ray-microscopes.html). Measurements were standardized using phantoms (i.e., 0.25 y 0.75 g/cm^3^) provided for bone density (CT-Analyzer software v1.13; https://www.bruker.com/en/products-and-solutions/microscopes/3d-x-ray-microscopes.html)^50,63^. Density measurements were randomly performed in 3 regions of the shell (a: newly growing edge; b: center; and c: umbo), representing a 3*D* volume of *ca.* 25µm^3^ per side. At the growing edge (ca. 1 × 1 mm), shell microhardness was measured using a Zwick hardness tester with a Vickers indenter (ASTM C1327-15). Shell samples were placed in 30 mm silicon molds, embedded in epoxy resin (12 h), then ground with silicon carbide paper (from 600 to 2400 grids) and polished with aluminum oxide suspensions (from 0.1 to 0.04 um). Vickers microhardness tests (8–9 random indentations, n = 4 shells per treatments, 1 per aquaria) were conducted at room temperature, applying a load of 2.94 N and dwell time of 10 s^17,22^. 400 × photographs were used to measure the diagonal of the imprint indented area using ImageJ software v.152a (https://imagej.nih.gov/ij/)^64^. Then, the Vickers Hardness (MPa) was calculated as HV = 0.1891 P/d^2^ where *P* is the applied load (N) and *d* the mean of the two measured diagonals (mm). Uniaxial compression tests were performed using a universal texturing machine (Instron 3342). The compression test uses shell fragment (2 × 2 mm) from the growing shell edge and oriented along three main planes of the shell fragments: longitudinal to the shell ribs; across the sagittal section (thickness) and transversal to the ribs. Tensile tests were also performed under dry and re-hydration conditions^56^, using synthetic seawater (INSTANT OCEAN) at 33 psu in salinity.

### Statistical analyses

In all cases shell properties measurements were performed on individual shells to avoid pseudo replication issues. Repeated measurements were done on scallop shells subjected to TGA, ATR, XRD analyses but analyzed separately using factorial ANOVAs including Tukey’s HSD as a posteriori test to evaluate the effects of pH and temperature treatments on organic matter in the shell carbonate matrix and organic content in shell periostracum. Differences in mineral density and micro-hardness were tested using a mixed factorial nested ANOVA, regarding shell areas (edge, center, and umbo within the same shell) and repeated indentations within shell as random effects, and temperature—pH treatments as fixed effects. Finally, using *r*–Pearson correlation index, we explored the paired relationship between organic matter and compounds recorded in the shell matrix and periostracum with physiological rates for exactly the same individual scallops^11,30^. ANOVAs and correlations were carried out using MINITAB v14 (https://www.minitab.com).

## Supplementary Information

Supplementary Information.

## Data Availability

Datasets generated and analyzed during the current study are available from the corresponding author.
