# Supplementary material for: Plasticity in organic composition maintains biomechanical performance in shells of juvenile scallops exposed to altered temperature and pH conditions
Source: Sci Rep. 2021 Dec 17;11:24201. doi: 10.1038/s41598-021-03532-0 (PMC8683433; doi:10.1038/s41598-021-03532-0)
Supplement: Supplementary file 1 — Supplementary Information. [file 41598_2021_3532_MOESM1_ESM.docx]

**Supplementary material**

**Plasticity in organic composition maintains biomechanical performance in shells of juvenile scallops exposed to altered temperature and pH conditions**

Nelson A. Lagos^1,2*^, Samanta Benítez^1,3^, Cristian Grenier^4^, Alejandro Rodriguez-Navarro^4^, Claudio García-Herrera^5^, Aldo Abarca-Ortega^5,6^, Juan F. Vivanco^7^, Isabel Benjumeda^8^, Cristian A. Vargas^9^, Cristian Duarte^10,11^ & Marco A. Lardies^2,8^

^1^Centro de Investigacion e Innovacion para el Cambio Climatico (CiiCC), Facultad de Ciencias, Universidad Santo Tomas, Santiago, Chile

^2^Instituto Milenio de Socio-Ecologia Costera (SECOS), Chile

^3^Programa de Doctorado en Biología Marina, Instituto de Ciencias Marinas y Limnologicas, Universidad Austral de Chile, Valdivia, Chile.

^4^Departamento de Petrologia y Mineralogia, Facultad de Ciencias, Universidad de Granada, España

^5^Departamento de Ingeniera Mecanica, Universidad de Santiago de Chile, Santiago, Chile.

^6^Centro de Tecnología Biomedica, Universidad Politecnica de Madrid, 28223, Pozuelo de Alcorcon, Madrid, España

^7^Facultad de Ingenieria & Ciencias, Universidad Adolfo Ibañez, Viña del Mar, Chile

^8^Facultad de Artes Liberales, Universidad Adolfo Ibañez, Santiago, Chile

^9^Laboratorio de Ecosistemas Costeros & Cambio Ambiental Global (ECCALab), Facultad de Ciencias Ambientales, Universidad de Concepción, Concepción, Chile.

^10^Departamento de. Ecología y Biodiversidad, Facultad de Ciencias de la vida, Universidad Andrés Bello, Santiago, Chile

^11^Centro de Investigación Marina Quintay (CIMARQ), Facultad de Ciencias de la Vida, Universidad Andrés Bello, Santiago, Chile

*Corresponding author at: Nelson A. Lagos. Facultad de Ciencias, Universidad Santo Tomás, Ejercito 146, Santiago, Chile, Email address: nlagoss@santotomas.cl

**Table S1.** Biological responses of *Argopecten purpuratus* individuals exposed to contrasting pH (7.7 and 8.0) and temperature (T) (14° and 18°C) levels in experimental treatments. Data synthetized from Lagos et al. 2016; Lardies et al. 2017.

| Biological response | pH~ 8.0 | | pH~ 7.7 | | Significant  effects |
| --- | --- | --- | --- | --- | --- |
|  | 14 ºC | 18ºC | 14ºC | 18ºC |  |
| Shell Thickness (mm) | 0.289 ± 0.031 | 0.194 ± 0.017 | 0.244 ± 0.016 | 0.178 ± 0.008 | T |
| Shell Height (mm d^-1^) | 0.109 ± 0.011 | 0.110 ± 0.004 | 0.068 ± 0.008 | 0.111 ± 0.005 | T × pH |
| Shell Length (mm d^-1^) | 0.184 ± 0.021 | 0.209 ± 0.014 | 0.111 ± 0.008 | 0.195 ± 0.009 | T + pH |
| Shell Dry Weight (g) | 6.29 ± 0.107 | 5.85 ± 0.109 | 5.935 ± 0.104 | 5.802 ± 0.103 | T |
| Shell Dissolution Rate (mg d^-1^) | 0.023 ± 0.004 | 0.019 ± 0.012 | 0.049 ± 0.013 | 0.035 ± 0.015 | T + pH |
| Net Calcification Rate (g d^-1^) | 0.009 ± 0.003 | 0.026 ± 0.001 | 0.016 ± 0.003 | 0.024 ± 0.001 | T |
| Biomass increase (g d^-1^) | 0.369 ± 0.016 | 0.362 ± 0.022 | 0.335 ± 0.009 | 0.342 ± 0.021 | pH |
| Growth Rate (g d^-1^) | 0.009 ± 0.003 | 0.026 ± 0.001 | 0.016 ± 0.003 | 0.024 ± 0.001 | T |
| Metabolic Rate (mgO_2_h^-1^g^-1^) | 0.052 ± 0.013 | 0.078 ± 0.008 | 0.066 ± 0.013 | 0.109 ± 0.010 | T |
| Ingestion Rate (mg chl h^-1^ g^-1^) | 1.481 ± 0.100 | 1.646 ± 0.064 | 1.920 ± 0.125 | 1.519 ± 0.139 | T × pH |


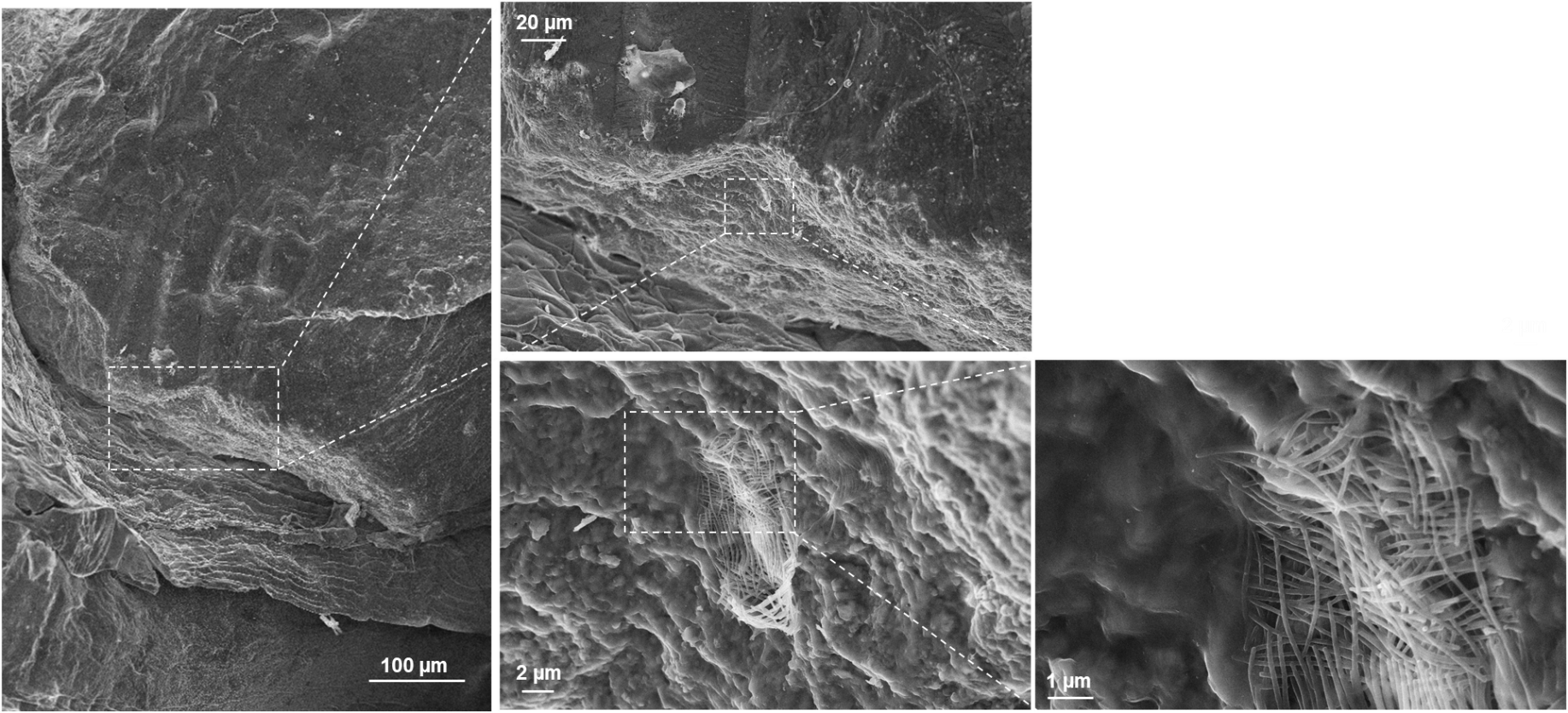


**Figure S1.** SEM observations on the outer surface of the newly shell growth margin of juvenile scallops *Argopecten purpuratus* after exposed to control conditions (temperature 14°C and pH level ~ 8.0). Note the abundant fibrous material over the shell surface.


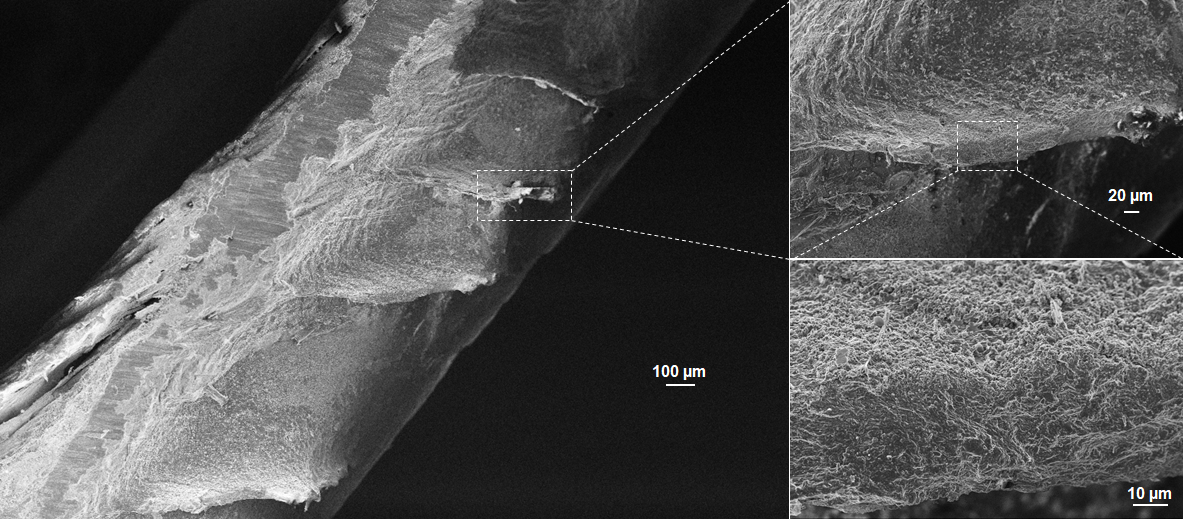


**Figure S2**. SEM observation on the outer surface of the juvenile scallops *Argopecten* *purpuratus* secondary microstructures along the shell ribs. Individual was exposed to acidified conditions (pH ~ 7.7) and control temperature (14°C). Note the absence of fibrous material and corrosion of the secondary structures deposited along the shell ribs.


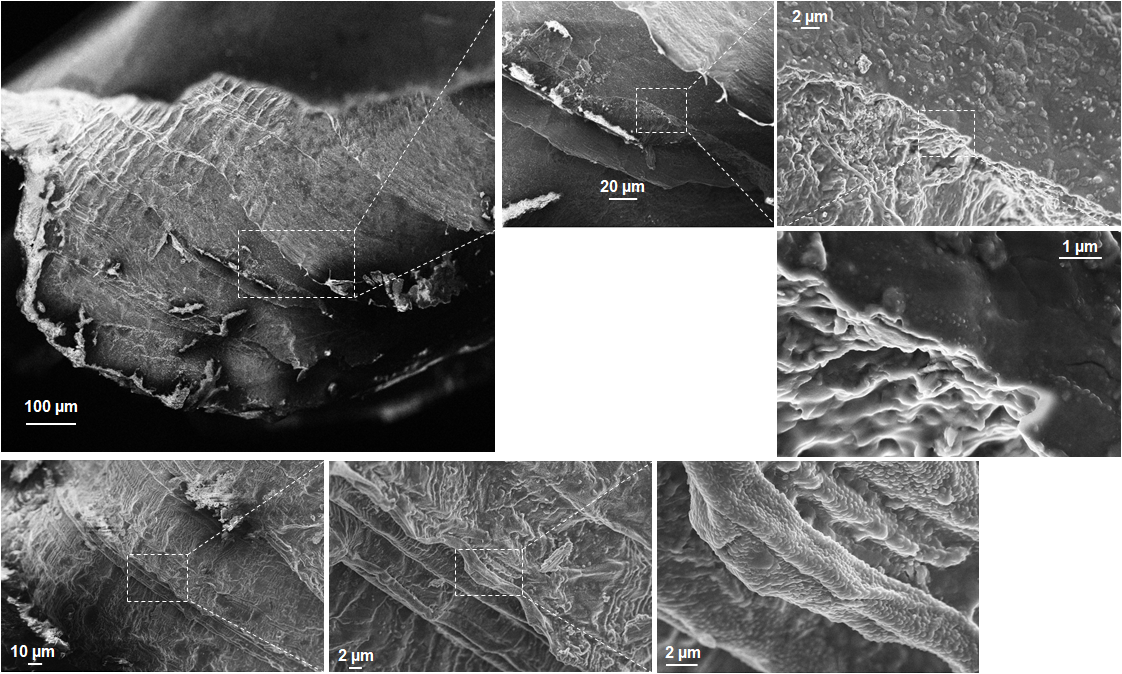


**Figure S3**. SEM observations on the outer surface of newly shell growth margin of juvenile scallops *Argopecten* *purpuratus* after exposed to control (pH ~ 8.0) and increased temperature (18°C). Note the globular structure of the periostracum covering the microstructures of the shell.

**
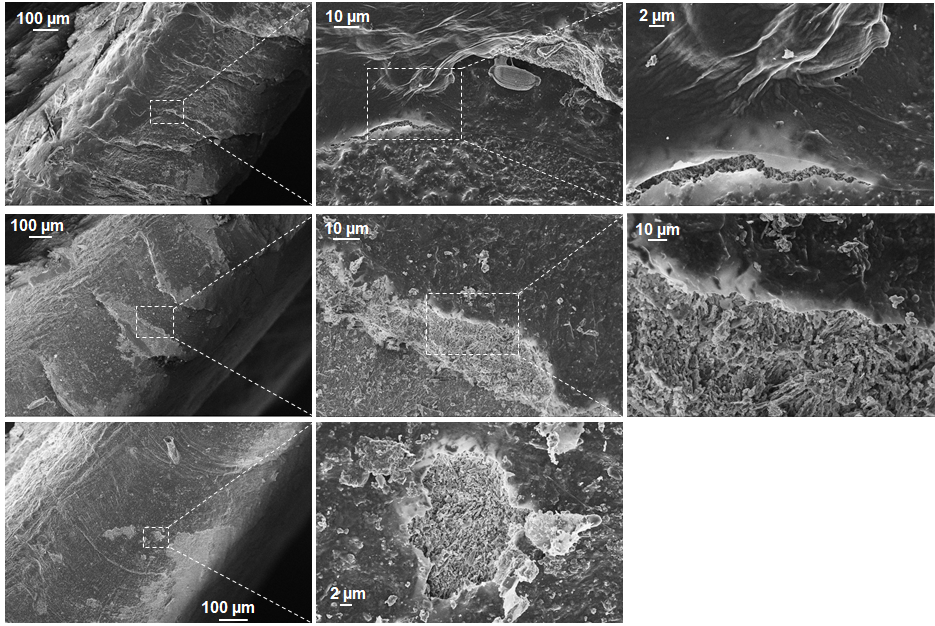
**

**Figure S4.** SEM observations on the outer Shell surface of the juvenile scallops *Argopecten* *purpuratus* after exposed to low pH (~ 7.7) and increased temperature (18°C) conditions. Note the presence of less notable secondary structures along the ribs and the erosion of the shell periostracum.
